# Spatial Metabolic Profiling of Human Atrial Tissue in Atrial Fibrillation Using DESI-MSI: An Exploratory Pilot Study

**DOI:** 10.1177/11795468261486227

**Published:** 2026-09-06

**Authors:** Beatrice Bacchi, Fabrizio Rosati, Camila Mayorga Palacios, Martin Kaufmann, Kevin Ren, John F. Rudan, Gianluigi Bisleri

**Affiliations:** 1 Division of Cardiac Surgery, St Michael’s Hospital, 7938 University of Toronto, Toronto, ON, Canada; 2 Division of Cardiac Surgery, ASST Spedali Civili di Brescia, University of Brescia, Italy; 3 Department of Biomedical and Molecular Sciences, 4257 Queen’s University, Kingston, ON, Canada; 4 Department of Pathology, 4257 Queen’s University, Kingston, ON, Canada; 5 Department of Surgery, Kingston Health Science Centre, Queen’s University, Kingston, ON, Canada

**Keywords:** atrial fibrillation, DESI-MSI, metabolomics, mass spectrometry imaging, atrial remodeling

## Abstract

**Background:**

Structural remodeling and atrial fibrosis are hallmarks of atrial fibrillation (AF), yet molecular alterations associated with atrial remodeling remain incompletely characterized, particularly their spatial distribution within human atrial tissue. Desorption Electrospray Ionization Mass Spectrometry Imaging (DESI-MSI) enables spatially resolved metabolic profiling of tissue sections and may provide insight into metabolic alterations associated with atrial remodeling in AF. This study investigated whether DESI-MSI could identify distinct metabolic signatures in atrial tissue from patients with AF.

**Methods:**

In this exploratory pilot study, atrial tissue samples from 10 patients undergoing cardiac surgery (4 with AF and 6 controls) underwent DESI-MSI analysis across an m/z range of 50–1200, followed by histologic assessment with hematoxylin–eosin staining. Metabolic profiles were analyzed using principal component analysis (PCA) and linear discriminant analysis (LDA) to explore group-specific metabolic patterns.

**Results:**

Histologic evaluation demonstrated minimal fibrosis without significant differences between AF and control samples. In contrast, DESI-MSI identified distinct metabolic features associated with AF. Arachidonic acid (m/z 303.2) and phosphatidylinositol species (m/z 885.5) demonstrated spatial correspondence with myocardial architecture and contributed to metabolic separation between groups. Exploratory supervised multivariate analysis showed partial separation between AF and control spectra, while LD loading plots identified differential lipid-related ion patterns associated with AF samples.

**Conclusions:**

In this exploratory pilot cohort, DESI-MSI demonstrated preliminary metabolic differences between AF and control atrial tissues despite limited histologic remodeling. These findings should be considered hypothesis-generating and will require validation in larger, adequately matched cohorts integrating metabolic, histologic, and clinical phenotypes. Although they do not establish that metabolic remodeling precedes fibrosis, they raise the possibility that DESI-MSI may detect metabolic differences in atrial tissue exhibiting minimal histologic remodeling.

Atrial fibrillation (AF) is the most common cardiac arrhythmia worldwide and is associated with substantial morbidity, mortality, and healthcare burden^1^

Despite advances in rhythm-control strategies, the mechanisms underlying AF initiation and progression remain incompletely understood.^2^ Structural remodeling, particularly atrial fibrosis, represents a major component of the arrhythmogenic substrate and contributes to electrical heterogeneity, impaired conduction, and AF persistence.^1,2^ Notably, atrial myocardium is more susceptible to fibrotic remodeling than ventricular tissue, a characteristic that correlates with increased recurrence rates, reduced response to antiarrhythmic therapies, and greater procedural complexity and complications.^3^ However, conventional histologic assessment may fail to capture the full molecular heterogeneity associated with atrial remodeling.

Increasing evidence suggests that metabolic dysregulation plays an important role in AF pathophysiology. Previous metabolomic studies have demonstrated alterations in myocardial energy metabolism, ketone body utilization, lipid metabolism, and mitochondrial function in patients with AF. These metabolic perturbations have also been associated with pathways involved in electrical and structural remodeling, supporting the concept that metabolic changes may contribute to the development and maintenance of the arrhythmogenic substrate.^4-7^

Nevertheless, direct spatial correlation between metabolic alterations and atrial tissue architecture remains limited.

Desorption Electrospray Ionization Mass Spectrometry Imaging (DESI-MSI) enables spatially resolved metabolic characterization of tissue sections with minimal sample preparation. By combining molecular and histologic information, DESI-MSI allows visualization of metabolite distribution within intact tissue architecture and has demonstrated utility in oncologic and cardiovascular tissue analysis. In cardiac research, DESI-MSI has shown potential for identifying disease-associated lipidomic patterns^8-12^; however, its application to human AF tissue remains largely unexplored.

The primary objective of this exploratory pilot study was to evaluate the feasibility of applying DESI-MSI to human atrial tissue and to explore whether spatial metabolic differences could be detected between atrial tissue from patients with and without AF. This study was designed as a hypothesis-generating feasibility investigation rather than to establish disease-specific metabolic signatures or diagnostic biomarkers.

## Methods

### Study Design and Tissue Collection

This exploratory, single-center pilot case–control study included 10 consecutive patients undergoing cardiac surgery. Clinical and demographic variables were collected from routine preoperative assessment. Atrial tissue samples were obtained intraoperatively from the right atrium before venous cannulation for cardiopulmonary bypass. Tissue collection did not require additional surgical manipulation beyond standard operative practice.

Patients in the AF group had a documented history of AF, whereas control patients had no prior history or documented episodes of AF and were in sinus rhythm at the time of surgery.

Given the pilot nature of the study and the availability of atrial tissue specimens, no formal sample-size calculation was performed.

### DESI-MSI Analysis

A total of 10 atrial tissue specimens (4 AF, 6 controls) were snap-frozen in an isopentane cryobath at −60°C and cryosectioned at a thickness of 10 μm. Unstained frozen sections underwent DESI-MSI analysis for lipid characterization over an m/z range of 50–1200.

DESI-MSI was performed using a Prosolia 2D DESI source coupled to a Xevo G2-XS quadrupole time-of-flight mass spectrometer (Waters Corporation, Milford, MA, USA) equipped with a heated inlet capillary maintained at 450°C. Imaging acquisition was performed in a pixel-by-pixel manner with a spatial resolution of 100 × 100 μm, with a mass spectrum acquired at each pixel to generate two-dimensional molecular ion maps allowing qualitative visual comparison of metabolite distribution with the corresponding H&E-stained section of the same tissue specimen.

Following DESI-MSI acquisition, the same tissue sections were stained with hematoxylin–eosin (H&E), allowing direct visual comparison between metabolite distribution and tissue morphology. No computational image registration or quantitative co-localization analysis was performed.

The resulting DESI-MSI datasets were processed for exploratory multivariate analysis using Principal Component Analysis (PCA) and Linear Discriminant Analysis (LDA).

Metabolite assignments should be considered putative and were based on accurate mass measurements, comparison with published literature, and spatial distribution patterns. Orthogonal validation by tandem mass spectrometry (MS/MS), authentic reference standards, or targeted lipidomic analyses was not performed.

### Histologic Assessment

Following DESI-MSI acquisition, tissue sections were stained with hematoxylin–eosin (H&E) and reviewed by an experienced pathologist blinded to clinical status. Histologic assessment included evaluation of myocytolysis, nuclear hypertrophy, interstitial lymphocytic inflammation, fibrosis, amyloid deposition, and endocardial thrombus.

Myocytolysis was defined as clearing involving at least 10% of the cytoplasm. Nuclear hypertrophy was categorized as absent/mild, moderate, or marked according to the proportion of enlarged nuclei relative to background myocytes. Interstitial lymphocytic inflammation was graded using a modified World Heart Federation classification.

Fibrosis was qualitatively categorized as absent/mild, moderate, or marked based on the extent of interstitial collagen deposition. Because of the limited availability of tissue and the experimental workflow, which prioritized DESI-MSI followed by H&E staining on the same tissue sections to preserve spatial correspondence, fibrosis-specific stains such as Masson’s trichrome or Sirius Red were not performed. Consequently, subtle interstitial fibrosis may not have been detected, limiting the ability to comprehensively assess the relationship between metabolic alterations and structural remodeling.

Histological assessment was performed qualitatively by an experienced pathologist blinded to clinical status and was not intended to provide quantitative image registration with DESI-MSI datasets.

### Statistical Analysis

Continuous variables are presented as mean ± standard deviation or median [interquartile range] according to data distribution. Categorical variables are expressed as counts and percentages. Group comparisons were performed using Student’s t-test, Mann–Whitney U test, or Fisher’s exact test, as appropriate. A two-sided P value <0.05 was considered statistically significant.

Exploratory multivariate analyses were performed using Principal Component Analysis (PCA) and Linear Discriminant Analysis (LDA) to identify overall metabolic patterns and visualize group separation. PCA was applied as an unsupervised dimensionality-reduction technique, whereas LDA was used to identify spectral features contributing to discrimination between the predefined groups. Given the exploratory pilot design and the very limited sample size, all statistical and multivariate analyses were considered descriptive and hypothesis-generating. They were not intended for predictive model development, formal biomarker identification, or definitive inference regarding differences between AF and control populations. Accordingly, p-values have not been provided.

## Results

### Patient Characteristics

Baseline clinical characteristics are summarized in Table 1. Patients with AF were older than controls (73.5 ± 4.6 vs. 63.7 ± 9.5 years), although this difference did not reach statistical significance (p = 0.06). Major cardiovascular comorbidities, including hypertension, diabetes mellitus, and dyslipidemia, were similarly distributed between groups.

**Table 1.** Patient Characteristics

​	AF	Control
Patients (n°)	4	6
Age, ys	73,5 ± 4,6	63,7 ± 9,5
Female sex	2/4 (50%)	1/6 (16.7%)
BMI (kg/m^2^)	27.6 ± 2.1	28.6 ± 2.5
Hypertension	3/4 (75%)	5/6 (83.3%)
Diabetes Mellitus	2/4 (50%)	2/6 (33.3%)
Dyslipidemia	2/4 (50%)	4/6 (66.7%)
Chronic Kidney Disease	2/4 (50%)	1/6 (16.7%)
eGFR (ml/min/1.73m^2^)	63.5 ± 26.3	78.7 ± 18.0
Vascular Disease	2/4 (50%)	0/6 (0%)
LVEF (%)	57.7 ± 18.4	63.3 ± 7.55

Patients with AF demonstrated a higher prevalence of chronic kidney disease (50% vs. 16.7%) and lower estimated glomerular filtration rate (eGFR) compared with controls. Body mass index did not differ between groups (27.6 ± 2.1 vs. 28.6 ± 2.5 kg/m^2^). Table S1.

### Histologic Findings

Histologic findings are summarized in Table 2. Overall, atrial tissue samples demonstrated limited structural remodeling. Interstitial fibrosis was predominantly categorized as absent/mild in both AF and control groups, with no significant between-group differences (p = 0.67). Moderate fibrosis was identified in one AF specimen and one control specimen, whereas no samples demonstrated marked fibrosis.

**Table 2.** Histological Findings

Histologic parameter	AF (n=4)	No AF (n=6)
**Myocytolysis**		
Absent/mild (%)	2 (50%)	3 (50%)
Moderate (%)	1 (25%)	2 (33%)
Marked (%)	1 (25%)	1 (17%)
**Nuclear hypertrophy**		
Absent/mild (%)	2 (50%)	2 (33%)
Moderate (%)	2 (50%)	4 (67%)
Marked (%)	0	0
**Interstitial lymphocytic inflammation**		
Absent/mild (%)	4 (100%)	6 (100%)
Moderate (%)	0	0
Marked (%)	0	0
**Interstitial fibrosis**		
Absent/Mild	3 (75%)	5 (80%)
Moderate	1 (25%)	1 (20%)
Marked	0	0
**Amyloid deposition (%)**	0	0
**Endocardial thrombus (%)**	0	0

Similarly, no substantial differences were observed in myocytolysis, nuclear hypertrophy, or interstitial lymphocytic inflammation between groups. Amyloid deposition and endocardial thrombus were absent in all specimens.

Histologic evaluation using H&E demonstrated limited histologic evidence of structural remodeling, with no apparent differences between AF and control samples.

### DESI-MSI Metabolic Profiling

**Figure 1.**
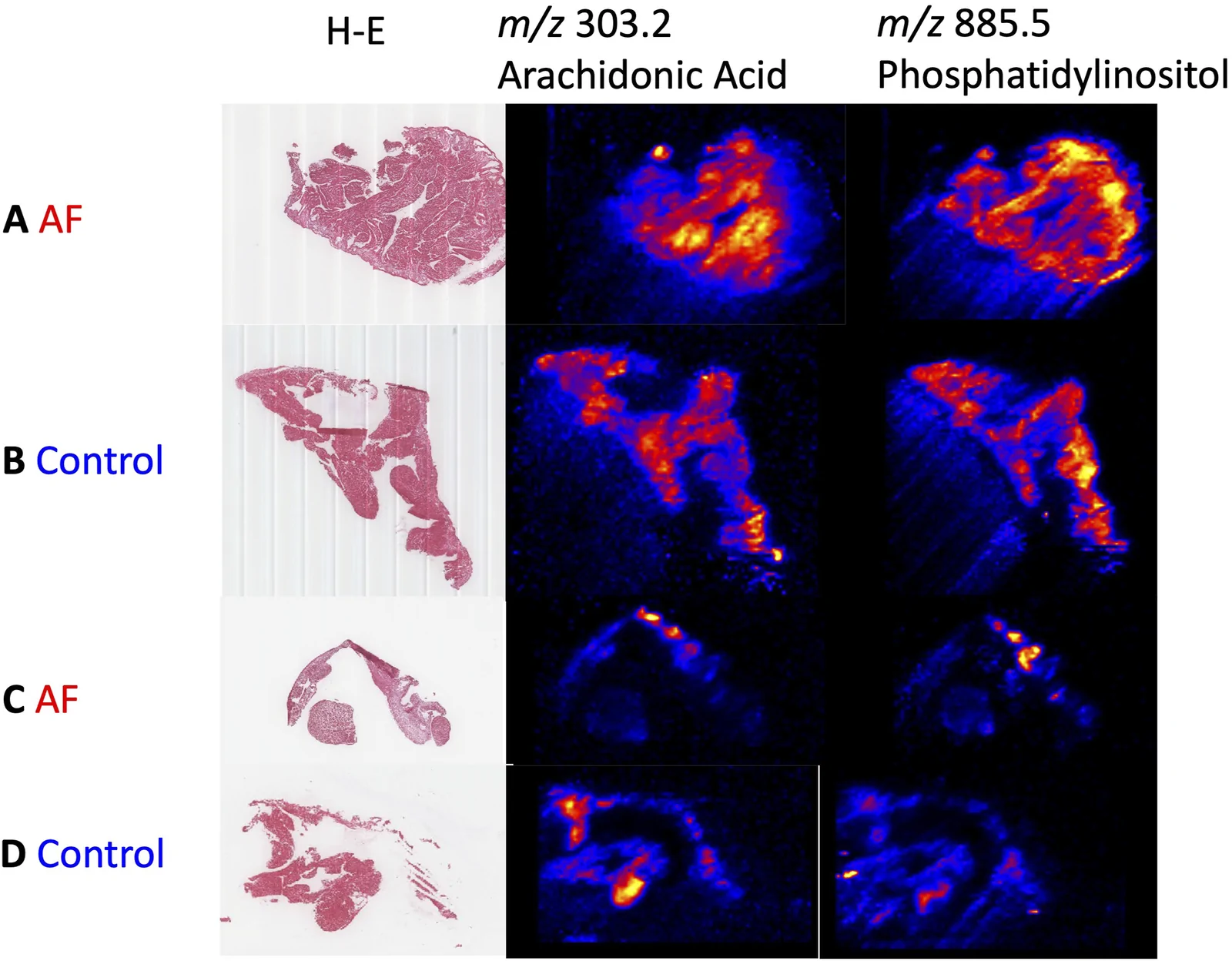
Spatial Distribution of Arachidonic Acid and Phosphatidylinositol Species in AF and Control Atrial Tissue. Representative H&E-stained sections and corresponding DESI-MSI ion images of atrial tissue from patients with and without AF. Rows A and C show representative samples from patients with AF, whereas rows B and D show representative control samples. For each specimen, the H&E section is shown alongside the spatial distribution of arachidonic acid (m/z 303.2) and phosphatidylinositol (m/z 885.5) obtained by DESI-MSI. Ion images are displayed using a heat scale in which blue indicates lower signal intensity and yellow/red indicates higher signal intensity, providing a qualitative visualization of metabolite signal distribution within atrial tissue. DESI-MSI images were acquired at a spatial resolution of 100 × 100 μm per pixel. The ion maps are presented as representative qualitative visualizations of metabolite signal distribution and are not intended for quantitative comparison of ion intensities between specimens or tissue compartments

Representative DESI-MSI ion images of arachidonic acid (m/z 303.2) and phosphatidylinositol species (m/z 885.5) provided a qualitative visualization of metabolite signal distribution relative to the underlying myocardial architecture on matched H&E sections (Figure 1). These ions are presented as representative examples of metabolite signal distribution and were not selected as the principal contributors to PCA/LDA group separation. Representative ion images illustrated the heterogeneous spatial distribution of metabolite signals across the tissue sections, providing a qualitative visualization of metabolite localization within atrial tissue.

### Multivariate Analysis of Metabolic Signatures

**Figure 2.**
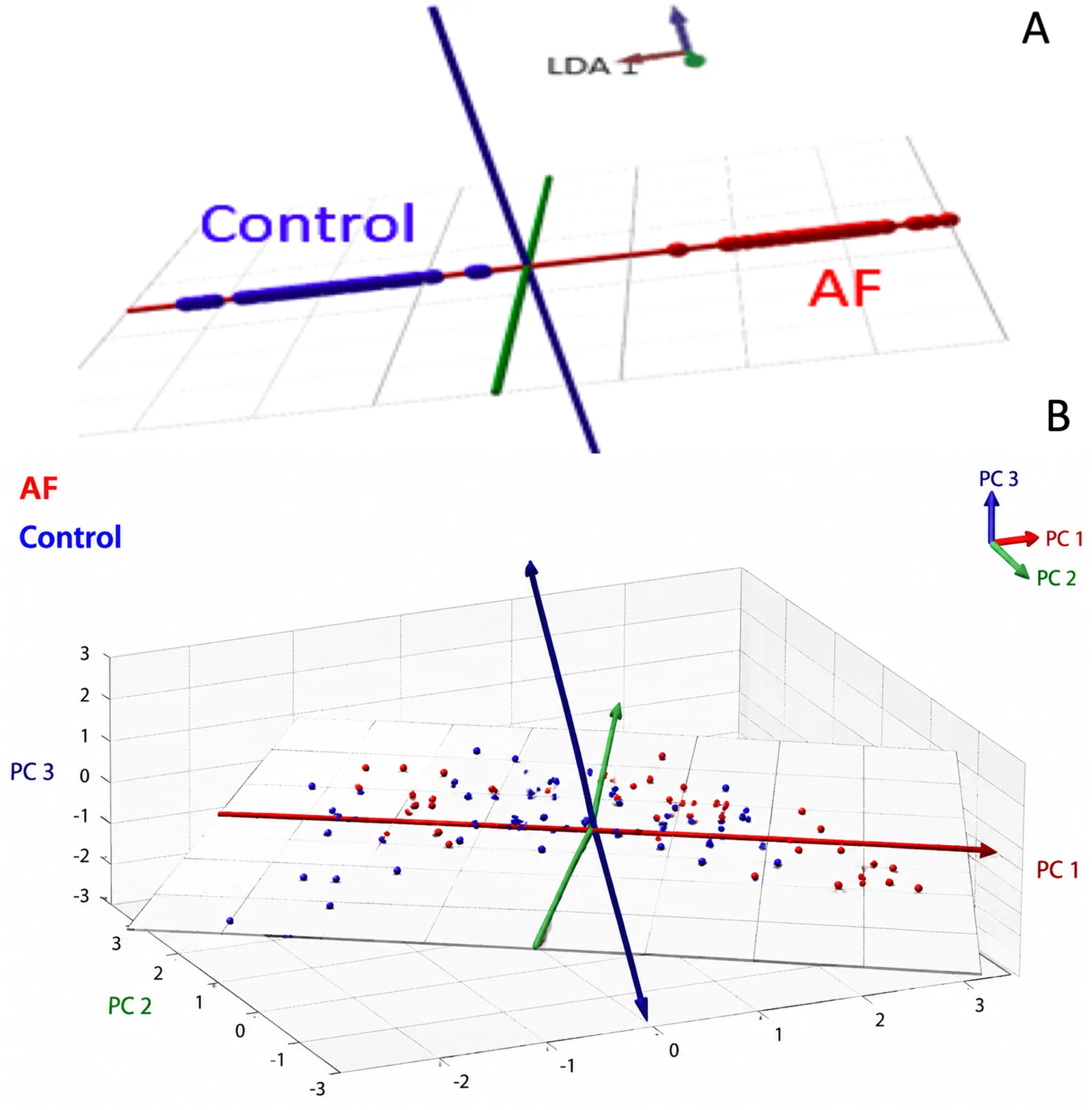
Exploratory multivariate analysis of DESI-MSI data from atrial tissue samples. (A) Two-dimensional Linear Discriminant Analysis (LDA) plot illustrating the separation between AF (red) and control (blue) groups along the primary discriminant axis. (B) Two-dimensional Principal Component Analysis (PCA) plot based on the first two principal components. The PCA shows partial clustering of AF and control data, with some overlap between groups, consistent with the exploratory nature of this pilot study

Exploratory multivariate analysis demonstrated metabolic differences between AF and control samples. Supervised LDA showed separation between AF and control spectra along the primary discriminant axis, whereas unsupervised PCA demonstrated partial clustering of samples according to clinical group (Figure 2).

**Figure 3.**
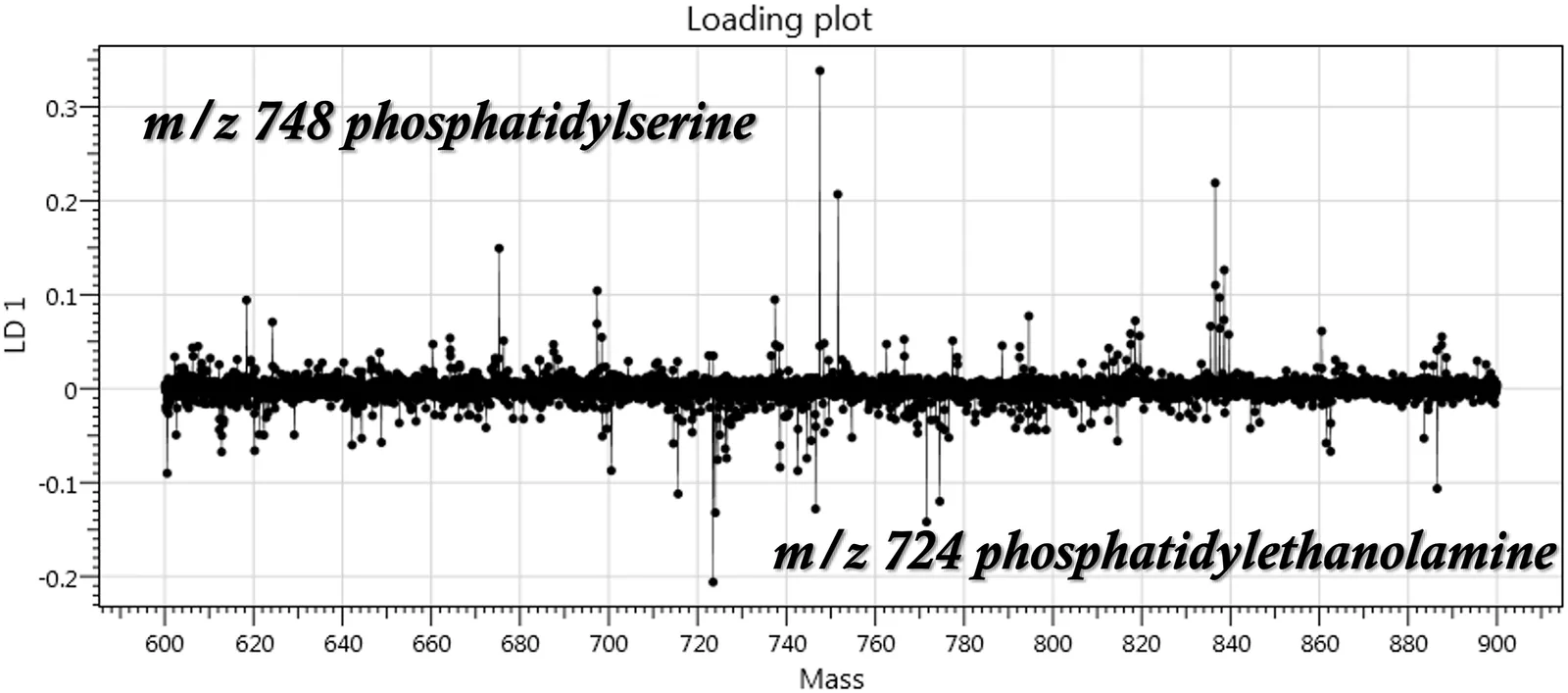
LD loading plot derived from exploratory Linear Discriminant Analysis (LDA). The LD loading plot illustrates the contribution of individual m/z features to discrimination between AF and control atrial tissue samples. Positive LD1 loading values indicate spectral features more strongly associated with the control group, whereas negative LD1 loading values indicate features more strongly associated with AF tissue. The highlighted ions at m/z 748 (putative phosphatidylserine) and m/z 724 (putative phosphatidylethanolamine) are presented as representative examples of discriminant spectral features identified by the loading plot. Metabolite annotations are putative and were assigned based on accurate mass measurements and comparison with published literature

The LD loading plot identified multiple ions contributing to the discrimination between groups. Positive LD1 loading values indicate spectral features more strongly associated with the control group, whereas negative loading values indicate features more strongly associated with AF tissue. Among these, ions around m/z 748 and m/z 724 are presented as representative examples of spectral features associated with the control and AF groups, respectively, and should not be interpreted as the exclusive or predominant contributors to the overall multivariate classification. Their metabolite assignments are putative and based on accurate mass measurements and comparison with published literature. (Figure 3).

## Discussion

In this exploratory pilot feasibility study of 10 patients, we evaluated the feasibility of applying DESI-MSI to human atrial tissue and observed preliminary differences in the spatial metabolic profiles of atrial tissue from patients with AF compared with controls. These observations were identified in specimens showing limited histologic evidence of structural remodeling, suggesting that DESI-MSI may detect metabolic differences despite minimal morphologic changes. However, the present study was not designed to determine whether these metabolic alterations precede, accompany, or follow structural remodeling. In addition, given the very small sample size, the clinical imbalance between groups, and the absence of external validation, these findings should be considered hypothesis-generating and should not be interpreted as evidence of a definitive AF-specific metabolic signature.

Histologic assessment demonstrated predominantly absent or mild fibrosis in both AF and control specimens, with moderate fibrosis identified in only one sample per group. Similarly, myocytolysis, nuclear hypertrophy, and inflammatory changes were limited across the cohort. In addition, subtle interstitial collagen deposition may have been underestimated since fibrosis-specific stains such as Masson’s trichrome or Sirius Red were not available for the frozen tissue sections used for DESI-MSI. Consequently, the apparent dissociation between metabolic alterations and structural remodeling should not be interpreted as evidence that metabolic remodeling necessarily occurs before fibrosis but rather as an observation requiring confirmation using dedicated fibrosis-specific histologic techniques. Despite these relatively subtle histologic findings, DESI-MSI revealed clear differences in tissue metabolic organization. In particular, arachidonic acid (m/z 303.2) and phosphatidylinositol species (m/z 885.5) demonstrated spatial correspondence with myocardial architecture on matched H&E sections and contributed to group-related metabolic separation.

Importantly, the present cohort should not be interpreted as representing patients with “early” AF. Detailed clinical information regarding rhythm status at the time of tissue collection, and atrial dimensions, was not uniformly available. Consequently, the limited H&E-detected fibrosis observed in these specimens should not be considered evidence of early disease but rather reflects the morphologic findings obtained with the histologic approach used in this study.

Exploratory multivariate analyses suggested differences in the distribution of metabolic features between AF and control spectra. Supervised LDA demonstrated separation between AF and control spectra along the primary discriminant axis, while unsupervised PCA showed partial clustering according to clinical group. Although exploratory and limited by sample size, these findings raise the possibility of differences in lipidomic organization between the sampled AF and control tissues. In addition, LD loading plots identified differential contributions of ions around m/z 724 and m/z 748 to AF and control samples, respectively, supporting the presence of disease-associated metabolic heterogeneity within atrial tissue.

Structural remodeling and fibrosis are recognized hallmarks of AF progression and contribute to electrical heterogeneity, impaired conduction, and arrhythmia maintenance.^1,2,13^ However, conventional histologic assessment may underestimate early molecular alterations preceding advanced tissue remodeling. Our findings are consistent with the growing recognition that metabolic dysregulation is associated with atrial remodeling in AF.^4-7^ However, because of the cross-sectional design and limited sample size, our data do not establish whether metabolic alterations precede structural remodeling or represent a consequence of the disease process.

Previous metabolomic studies have demonstrated alterations in energy metabolism, mitochondrial pathways, ketone utilization, and lipid metabolism in patients with AF.^4,6,7^ Several imaging and metabolomic platforms, including nuclear magnetic resonance spectroscopy, positron emission tomography, matrix-assisted laser desorption ionization (MALDI), and DESI-MSI, have been applied to characterize cardiovascular metabolic remodeling. While these techniques provide complementary metabolic information, DESI-MSI offers the advantage of spatially resolved molecular characterization with minimal tissue preparation, enabling direct integration of metabolic and histologic information within the same specimen.^8,9^

Previous cardiovascular applications of DESI-MSI have demonstrated its ability to characterize metabolic alterations in myocardial infarction and atherosclerotic tissue, supporting its translational potential in cardiac disease characterization.^9,10^ In addition, altered myocardial lipid accumulation patterns have been associated with cardiomyopathic remodeling processes using mass spectrometry–based imaging approaches.^14^

Among the lipid species evaluated in the present study, phosphatidylinositol species are of particular interest because of their role in intracellular calcium regulation through inositol triphosphate-mediated sarcoplasmic reticulum calcium release, a mechanism closely linked to atrial arrhythmogenesis. Similarly, arachidonic acid metabolism has been associated with inflammatory signaling, oxidative stress, and myocardial remodeling in several cardiovascular disease states.^15^

M/z 303.2 was putatively assigned to arachidonic acid, a lipid mediator involved in inflammatory signaling, oxidative stress, and myocardial remodeling. The signal at m/z 885.5 was putatively assigned to PI(38:4), an abundant phosphatidylinositol species involved in phosphoinositide signaling and intracellular calcium regulation. In addition, ions around m/z 724 and m/z 748, which contributed to the exploratory LD separation, were provisionally consistent with phosphatidylethanolamine- and phosphatidylserine-related species, respectively. These assignments are based on accurate mass and comparison with published lipidomic data and were not confirmed by MS/MS or authentic standards; they should therefore be regarded as tentative.

Although these lipid-related signals were not exclusive to AF tissue, their differential spatial distribution and contribution to multivariate clustering support the biologic plausibility of the observed metabolic signatures. These mechanistic considerations are speculative and are presented only to provide a biological context for the putative metabolite assignments.

These metabolites are widely distributed in mammalian tissues and should not be interpreted as AF-specific molecular markers. Rather, their contribution to the exploratory multivariate separation supports the presence of differences in tissue-level lipidomic organization that require confirmation using orthogonal metabolomic approaches.

An important consideration when interpreting these findings is the clinical imbalance between the AF and control groups. Although most baseline characteristics were comparable, patients with AF exhibited lower renal function and a higher prevalence of chronic kidney disease. Renal dysfunction is known to influence systemic lipid metabolism and may also affect tissue metabolic composition. Therefore, we cannot exclude the possibility that some of the observed metabolic differences reflect, at least in part, differences in renal function or other baseline clinical characteristics rather than AF itself. Future studies, including larger, clinically matched cohorts and multivariable adjustment for potential confounders, will be necessary to better define AF-specific metabolic alterations.

Although DESI-MSI demonstrated measurable metabolic differences in this exploratory cohort, these observations should be interpreted cautiously, given the small sample size and the clinical imbalance between study groups. DESI-MSI identified metabolic differences in atrial tissue exhibiting limited histologic remodeling, suggesting that spatial metabolomic imaging may detect molecular heterogeneity beyond conventional histologic assessment. However, these findings do not establish a temporal relationship between metabolic remodeling and fibrosis. Rather, they highlight the potential utility of DESI-MSI as a translational tool for tissue-level characterization of atrial remodeling and warrant further investigation in larger, well-matched cohorts.

## Limitations

This study has several limitations. First, this was a very small exploratory pilot study including only 10 patients, of whom 4 had AF. The limited number of biologically independent patient samples substantially restricts statistical power, generalizability, and the ability to account for clinical confounding. The observed separation of spectra should therefore be interpreted as preliminary and hypothesis-generating rather than as evidence of a validated AF-specific metabolic phenotype. The PCA and LDA analyses were used only to explore patterns within this dataset and were not intended to develop or validate a predictive classification model. Confirmation in larger, independently recruited and adequately matched cohorts is required.

Second, the AF and control groups were not perfectly matched with respect to baseline clinical characteristics. In particular, patients with AF had lower renal function and a higher prevalence of chronic kidney disease. Because renal dysfunction may influence both systemic and tissue metabolic profiles, we cannot exclude that part of the observed metabolic differences may be attributable to renal impairment or other unmeasured clinical confounders rather than atrial fibrillation itself. Given the limited sample size, adjustment for these potential confounding factors was not feasible. Future studies should include larger, clinically matched cohorts to better isolate metabolic alterations specifically associated with AF.

Third, histologic evaluation was performed on frozen tissue sections, precluding the use of fibrosis-specific stains such as Masson’s trichrome or Sirius Red. Consequently, subtle interstitial fibrosis may have been underestimated, limiting our ability to accurately characterize the extent of structural remodeling and to directly relate metabolic findings to tissue fibrosis. Future studies integrating DESI-MSI with dedicated fibrosis-specific histologic assessment will be important to better define the relationship between metabolic alterations and structural remodeling in atrial fibrillation.

Moreover, detailed AF phenotyping, including rhythm status at tissue collection, atrial dimensions, and prior rhythm-control therapies, was not available. Therefore, the relationship between the observed metabolic profiles and the clinical stage of AF could not be assessed.

In addition, the study groups were not sex-matched, with a higher proportion of female patients in the AF group than in the control group. Given the very limited sample size, the potential influence of sex on atrial metabolic profiles could not be evaluated. Future studies including larger, sex-balanced cohorts, will be necessary to assess potential sex-related metabolic differences.

Finally, metabolite assignment was based primarily on mass-to-charge (m/z) features and spatial distribution patterns. Orthogonal validation by tandem mass spectrometry (MS/MS), authentic reference standards, or targeted lipidomic analyses was not performed. Consequently, the biological interpretation of the reported metabolite assignments should be considered preliminary and requires confirmation in future studies using complementary analytical approaches.

## Conclusions

In this small exploratory pilot cohort, DESI-MSI demonstrated preliminary differences in spatial metabolic profiles between AF and control atrial tissues. These observations should be considered hypothesis-generating and do not establish a validated or AF-specific metabolic signature. Larger, well-matched studies with independent validation are required to determine whether the observed metabolic patterns are reproducibly associated with AF.

DESI-MSI may provide a useful platform for future studies integrating spatial metabolic and histologic information within atrial tissue. However, its potential role in biomarker discovery, phenotypic stratification, or clinical characterization of AF remains to be established. Future investigations combining DESI-MSI with fibrosis-specific histologic staining and complementary molecular analyses will be essential to validate and extend these preliminary observations.

The present findings should not be interpreted as evidence that metabolic remodeling precedes fibrosis but rather as preliminary observations supporting further investigation of spatial metabolomic alterations in AF. Overall, these findings support the feasibility of applying DESI-MSI to human atrial tissue and provide a rationale for larger hypothesis-driven studies.

## Supplemental Material

Supplemental Material - Spatial Metabolic Profiling of Human Atrial Tissue in Atrial Fibrillation Using DESI-MSI: An Exploratory Pilot Study Supplemental Material for Spatial Metabolic Profiling of Human Atrial Tissue in Atrial Fibrillation Using DESI-MSI: An Exploratory Pilot Study by Beatrice Bacchi, Fabrizio Rosati, Camila Mayorga Palacios, Martin Kaufmann, Kevin Ren, John F. Rudan and Gianluigi Bisleri in Clinical Medicine Insights: Cardiology.

## Data Availability

The datasets generated during and/or analyzed during the current study are available from the corresponding author on request.*
