# Supplementary material for: Spatial Metabolic Profiling of Human Atrial Tissue in Atrial Fibrillation Using DESI-MSI: An Exploratory Pilot Study
Source: Clin Med Insights Cardiol. 2026 Sep 6;20:11795468261486227. doi: 10.1177/11795468261486227 (PMC13554659; doi:10.1177/11795468261486227)
Supplement: Supplementary file 1 [file sj-pdf-1-cic-10.1177_11795468261486227.pdf]

Supplementary Table S1. Individual patient-level clinical characteristics

All tissue specimens were harvested from the right atrium before venous cannulation for cardiopulmonary bypass.

AF status	AF type	AF duration (months)	Age	Sex	Primary cardiac diagnosis	Surgery type	Comorbidities	eGFR (mL/min/1.73m2)
Control	None	NA	70	F	CAD	CABG	HTN - T2DM – dyslipidemia - ex smoker - fibromylagia	90
Control	None	NA	51	M	CAD	CABG	HTN - esinophilic esophagitis	94
AF	Paroxysmal	1	77	F	CAD	CABG	T2DM - Dyslipidemia - ex smoker	33
AF	Paroxysmal	2	76	M	CAD + AS	CABG+ AVR	HTN - T2DM - Dyslipidemia	94
Control	None	NA	78	M	CAD	CABG	None	84
AF	Paroxysmal	10	69	F	AS	Isolated AVR	HTN - ex smoker	53
AF	Persistent	5	80	M	CAD	CABG	HTN	74
Control	None	NA	61	M	CAD	CABG	HTN - ex smoker	67
Control	None	NA	70	M	CAD	CABG	Dyslipidemia - ex smoker	44
Control	None	NA	60	M	CAD	CABG	HTN - T2DM - Dyslipidemia - ex smoker	93

Abbreviations:

AF, atrial fibrillation; CAD, coronary artery disease; AS, aortic stenosis; CABG, coronary artery bypass grafting; AVR, aortic valve replacement; HTN, hypertension; T2DM, type 2 diabetes mellitus;
